# Pool-hmm: a Python program for estimating the allele frequency spectrum and detecting selective sweeps from next generation sequencing of pooled samples

**DOI:** 10.1111/1755-0998.12063

**Published:** 2013-01-11

**Authors:** Simon Boitard, Robert Kofler, Pierre Françoise, David Robelin, Christian Schlötterer, Andreas Futschik

**Affiliations:** 1 Laboratoire de Génétique Cellulaire, INRA24 Chemin de Borde Rouge, Auzeville CS 52627, Castanet Tolosan Cedex, 31326, France; 2 Institut für Populationsgenetik, Vetmeduni ViennaVeterinärplatz 1, Wien, A-1210, Austria; 3 Institute of Statistics and Operations Research, University of ViennaUniversitätsstrasse 5/9, Wien, A-1010, Austria

**Keywords:** allele frequency spectrum, hidden Markov models, next generation sequencing, pooled DNA, selective sweeps.

## Abstract

Due to its cost effectiveness, next generation sequencing of pools of individuals (Pool-Seq) is becoming a popular strategy for genome-wide estimation of allele frequencies in population samples. As the allele frequency spectrum provides information about past episodes of selection, Pool-seq is also a promising design for genomic scans for selection. However, no software tool has yet been developed for selection scans based on Pool-Seq data. We introduce Pool-hmm, a Python program for the estimation of allele frequencies and the detection of selective sweeps in a Pool-Seq sample. Pool-hmm includes several options that allow a flexible analysis of Pool-Seq data, and can be run in parallel on several processors. Source code and documentation for Pool-hmm is freely available at https://qgsp.jouy.inra.fr/.

## Introduction

The detection of genomic regions that evolved under natural selection is an important topic in population genetics. The case of hard sweeps, where a new mutant goes to fixation in a population due to strong directional selection, has received particular attention (Kim & Stephan 2002; Nielsen *et al*. 2005; Jensen *et al*. 2007; Boitard *et al*. 2009; Alachiotis *et al*. 2012).

The advent of next generation sequencing (NGS) technologies provides a new dimension to such genome scans for selection. In spite of the considerable reduction in the cost of sequencing, the sequencing of individuals on a population scale remains expensive. However, hard sweeps can be detected using only the sample allele frequencies along the genome, and this information can be obtained by sequencing DNA from a pool of individuals (Pool-Seq). Although Pool-Seq is considerably cheaper than the sequencing of individuals, there are some methodological challenges associated with the analysis of the resulting data. First, the reads covering a given position of the reference genome arise from a random sampling among the pooled chromosomes, so observations can be redundant. Second, sequencing error probabilities are larger than with classic Sanger sequencing (Luo *et al*. 2011) and are variable among and within reads.

Recently, Boitard *et al*. (2012) proposed a hidden Markov model (HMM) for detecting sweeps based on Pool-Seq data. This method involves computing the likelihood of the observed read information conditional on allele counts in the pool, for each genome position. Downstream analyses—estimation of the background allele frequency spectrum (AFS) and detection of selective sweeps—are then based on these likelihoods. Uncertainty concerning the true allele frequencies in the pool, which might typically be higher for sites with low coverage or bad quality scores, is thus taken into account in the analyses. Possible biases arising from unequal DNA concentration or quality among individuals are not accounted for by this method, but these effects are expected to be limited for large sample sizes (Futschik *et al*. 2010).

In this work, we propose a Python program, denoted Pool-hmm, that implements the method of Boitard *et al*. (2012). The two main applications of this program are AFS estimation and detection of selective sweeps, in a given region. These two applications are implemented independently, so it is possible, for instance, to detect selective sweeps based on a background AFS that is specified by the user. In addition, Pool-hmm provides an estimation of allele frequencies at each genomic position, which can be used in other population genetics software. These model-based estimations are preferable to naïve estimates obtained by computing the ratio of allele counts at a position, as discussed below.

## Input data

The main input of the program is the Pool-Seq data, which must be provided in the SAMtools pileup format (Li *et al*. 2009). Any alignment file in BAM or SAM format can easily be converted to the pileup format using the *samtools mpileup* command (without *-u* or *-g* options), independently of the software used to align or preprocess the reads.

## Allele frequency estimation

The method assumes an infinite sites model, where at most two alleles can be observed at each genomic position, the ancestral allele and the derived allele. By default, the ancestral allele is considered unknown, and Pool-hmm focuses on folded (rather than derived) allele frequencies. Two other strategies might also be specified (option *—ancestral-allele*). First, the reference allele provided in the pileup can be considered to be the ancestral allele. Second, ancestral alleles at each genomic position can be provided in an additional column of the pileup file.

For a given genomic region, Pool-hmm estimates the derived or folded AFS using an expectation maximization (EM) algorithm. The starting value for this EM is the expected AFS under a model with constant population size and scaled mutation rate θ = 4 *N*μ = 0.005, which can be modified by the user (option *–theta*).

Pool-hmm can also estimate the derived (or minor) allele frequency at each position in a specified region (option*—estim*). For this estimation, the previously estimated AFS (or any other AFS provided by the user) is considered as a prior. At a given position, it is combined with the likelihood of the observed reads to obtain a posterior distribution of the derived (or minor) allele frequency. The estimated frequency is the one maximizing this posterior distribution. This estimation procedure is more reliable than a direct estimation based on the ratio of allele counts at a position because it accounts for additional properties of the read data, namely the coverage and the base qualities at a given position. For instance, low-quality base calls have less influence on the allele frequency estimation. In addition, the estimated allele frequency at genomic positions with low coverage is essentially determined by the prior AFS.

Our likelihood-based approach for estimating the AFS in a region or allele frequencies at each genomic position is an alternative to discarding base calls or genomic positions based on arbitrary thresholds, and has the advantage of using the available information in a more continuous way. One important implication is that we can estimate without bias the proportion of singletons or other low allele counts, even at low (down to 0.5 × ) per chromosome coverage (Boitard *et al*. 2012), which is clearly not possible when thresholding genomic positions based on the number of alternative alleles.

Pool-hmm can be also used to compare the AFS of genomic regions with different annotations (e.g. introns vs. exons). We provide a script that filters the input pileup file for any feature present in an annotation .gtf file. Pool-hmm then infers the AFS based on the filtered pileup.

**Fig. 1:**
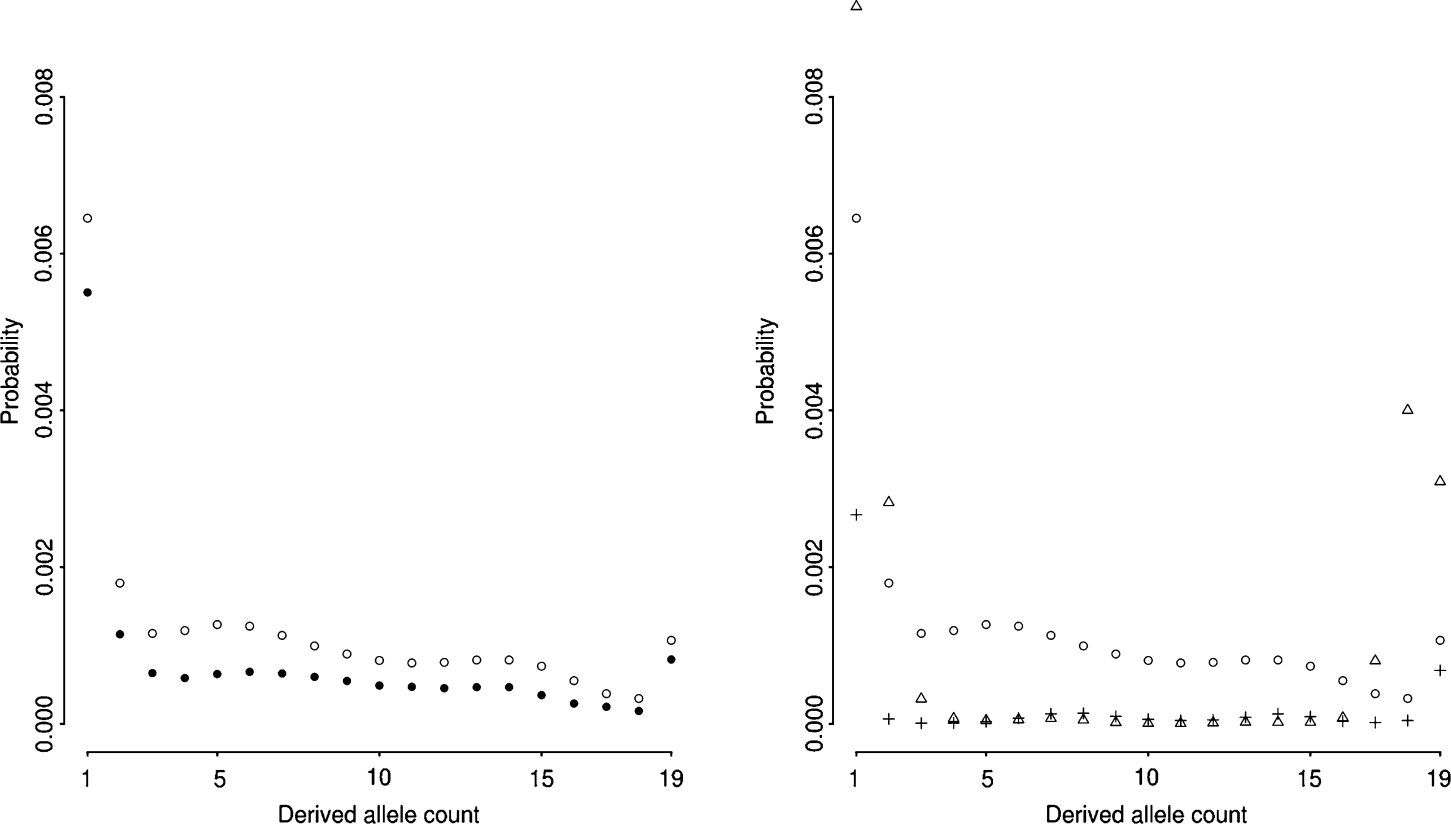
AFS in a quail sample of *n* = 20 chromosomes, computed from a random sample of genomic positions (empty circles), genomic positions within exons (full circles, left panel), genomic positions within sweep window 1 (empty triangles, right panel) or genomic positions within sweep window 2 (plus, right panel). Probabilities of 0- and 20-derived alleles are not shown because they are not at the same scale. The large probability observed for 19-derived alleles may be due to the misspecification of the ancestral allele at a small proportion of segregating sites. Such errors are expected if there is shared polymorphism between quail and chicken.

Note that the allele frequencies considered by Pool-hmm are sample allele frequencies (from 0 to *n*) and not population allele frequencies (from 0 to 1), see for instance the AFS in Fig. 1. Population and sample frequencies are closely related and essentially provide the same information, but inference based on coalescent theory, as the derivations of Nielsen *et al*. (2005) that are used in our sweep detection model, naturally involve sample allele frequencies.

## Detection of selective sweeps

In the HMM of Boitard *et al*. (2012), each genomic position is assumed to have a hidden state, which can take one of the three following values: ‘Selection’, for the sites that are very close to a swept site, ‘Neutral’, for the sites that are far away from any swept site and ‘Intermediate’ for the sites in between. These three values are associated with different AFS. The ‘Neutral’ AFS corresponds to the background (whole genome) AFS of the population. It can either be estimated from the Pool-Seq data or provided using option *—spectrum-file*. The ‘Intermediate’ and ‘Selection’ AFS are then deduced from the ‘Neutral’ AFS using the derivations in Nielsen *et al*. (2005), and are typically more skewed towards low and high allele frequencies. The hidden states form a Markov chain along the genome with a per-site probability *q* of switching state (argument *–k*). The observed variable at each genomic position is a vector summarizing the information provided by reads at this position.

In the HMM described above, a selective sweep is detected if the hidden state ‘Selection’ is inferred for a window of sites. Using Pool-hmm, this inference (option *—pred*) relies on two different criteria. First, the sequence of hidden states maximizing the likelihood of the HMM is computed using the Viterbi algorithm (Rabiner 1989) and returned in a file with suffix *.pred*. A summary of the sweep windows detected from this algorithm (genomic regions with predicted hidden state ‘Selection’) is also returned in a file with suffix *.stat*. Second, the posterior probability of hidden state ‘Selection’ is computed for each genomic position using the forward–backward algorithm, and is returned in a file with suffix *.post*.

## Parallelization

Analysis of whole genome NGS data can be very time-demanding. To speed up the execution of Pool-hmm, we parallelized the parts of the code where the likelihood of the observed read information conditional on the number of derived alleles in the pool is computed. These computations represent the largest computational cost (other algorithms, such as EM or Viterbi, are very fast in comparison), but they can be performed independently for each genomic position. Taking advantage of this property, our strategy is thus to build a queue of 10 kb blocks that are distributed for analysis to different processors. The management of this queue and the coordination of all processors are implemented using the Python *multiprocessing* library.

This parallelization strategy enables an optimized use of multiple processors on a single machine. When running Pool-hmm on a computer cluster, we also recommend cutting the whole genome data into large regions (typically chromosomes) and analysing these regions independently on different nodes. This can be combined with parallelization within each node, as described above.

## Example

We applied Pool-hmm to a sample of 10 quails that were sequenced in a single pool at 20 ×coverage. Reads were aligned against the chicken genome release *WUGSC2.1* using glint (Courcelle *et al*. 2008) and converted to pileup format using samtools (Li *et al*. 2009). We focused on chromosome 1 (≍200-Mb long, with 20 million observed segregating sites) and conducted the analyses described above. We used option *—a ‘reference’*, thereby assuming that quail ancestral alleles are those that are found on the chicken reference genome. The execution time of all Pool-hmm commands with one, four or eight processors is given in Table 1. As expected, it decreases significantly when the number of processors increases, although not linearly because some parts of the code, as for instance the queue management, are not parallelized. Using eight cores, a standard analysis involving AFS estimation and sweep detection took about 5 h. Additional sweep analyses with a different sensitivity (parameter -k) are then very fast because they can use intermediate results (the HMM emission probabilities at each observed segregating site) that are stored from the first analysis. Note also that AFS estimation is much faster than allele frequency estimation because it is based on only 2% of the genomic positions of chromosome 1 (chosen at random). The proportion of genomic positions used for AFS estimation can be defined using option *—ratio*.

**Table 1:** Execution time of Pool-hmm for the analysis of chromosome 1 in a quail sample of *n* = 20 chromosomes. Results are provided for several types of analyses and for one, four or eight available processors on a computing cluster. Pool-hmm commands corresponding to these analyses in the case of one available processor are listed below the table

Number of processors	AFS estimation*	First sweep prediction†	Additional sweep prediction‡	Allele frequency estimation§
1	6 h 4 min 9 s	12 h 21 min 36 s	0 h 10 min 14 s	31 h 7 min 47 s
4	1 h 57 min 46 s	4 h 32 min 57 s	0 h 09 min 21 s	7 h 47 min 9 s
8	1 h 33 min 30 s	3 h 15 min 53 s	0 h 07 min 06 s	4 h 17 min 14 s

* Python pool-hmm.py –input-file quail -n 20 -a ‘reference’ –only-spectrum –theta 0.005 –ratio 50.

† Python pool-hmm.py –input-file quail -n 20 -a ‘reference’ –pred –spectrum-file quail –k 0.0000000001.

‡ Python pool-hmm.py –input-file quail -n 20 -a ‘reference’ –pred –emit-file –k 0.0000000001.

§ Python pool-hmm.py –input-file quail -n 20 -a ‘reference’ –estim –spectrum-file quail.

The AFS on chromosome 1 is shown in Fig. 1. For comparison, Fig. 1 also shows the AFS obtained using only genomic positions located within exons (as the number of these positions is much smaller, we used all of them rather than only 2%). We filtered the pileup with Pool-hmm, using a gtf file corresponding to the latest Ensembl annotation of the chicken assembly used for the alignment (url:http://ftp://ftp.ensembl.org/pub/release-68/gtf/gallus_gallus/). Exonic regions have an overall deficit of segregating sites, but apart from that the shape of the AFS in these regions is close to that obtained from random regions.

Seventy-four sweep windows were detected on chromosome 1. The *.stat* file reporting these regions is provided as supporting information. The evidence for each sweep window can be assessed using the third column of this file, which represents the maximum of the posterior probability of hidden state ‘Selection’ along the window (in log scale). To illustrate the specificity of the sweep windows detected by our approach, we estimated the AFS in the sweep windows corresponding to the two first lines of the *.stat* file (Fig. 1, right panel). Sweep window 1 was characterized by an excess of low- and high-frequency alleles, whereas sweep window 2 was characterized by a general deficit of segregating sites. Indeed, the detection method implemented in Pool-hmm makes use of both the density of segregating sites and the allele frequency pattern among segregating sites to distinguish sweep regions from neutral regions. Further details on this point and comparisons with alternative approaches can be found in Boitard *et al*. (2009, 2012).

## Conclusion

Pool-hmm is the first software tool for Pool-Seq data that provides a probabilistic allele frequency estimation and detects selective sweeps on a genomic scale. The implemented statistical algorithms account for two important features of Pool-Seq data, the random sampling among chromosomes within the pool and sequencing errors. Pool-hmm includes several options that allow a flexible analysis of Pool-Seq data.

## Software availability

Source code and documentation for Pool-hmm is freely available at https://qgp.jouy.inra.fr/. Several test data sets are also provided.
